# Split-Ring Resonator Based Sensor for the Detection of Amino Acids in Liquids

**DOI:** 10.3390/s23020645

**Published:** 2023-01-06

**Authors:** Kirsten J. Dehning, Moritz Hitzemann, Alexander Gossmann, Stefan Zimmermann

**Affiliations:** Institute of Electrical Engineering and Measurement Technology, Department of Sensors and Measurement Technology, Leibniz University Hannover, Appelstr. 9A, 30167 Hannover, Germany

**Keywords:** amino acids, split-ring resonator, SRR, resonance frequency, relative permittivity, conductivity

## Abstract

Amino acids belong to the most important compounds for life. They are structural components of proteins and required for growth and maintenance of cells. Essential amino acids cannot be produced by the organism and must be ingested through the nutrition. Therefore, the detection of amino acids is of great interest when analyzing cell culture media and nutrition. In this work, we present a split-ring resonator as a simple but sensitive detector for amino acids. Split-ring resonators are RLC resonant circuits with a split capacitance and thus a resonance frequency that depends on the electromagnetic properties of a liquid sample at the split capacitance. Here, the split capacitance is an interdigital structure for highest sensitivity and covered with a fluidic channel for flow through experiments. First measurements with a vector network analyzer show detection limits in the range from 105 µM for glutamic acid to 1564 µM for isoleucine, depending on the electromagnetic properties of the tested amino acids. With an envelope detector for continuous recording of the resonance frequency, the split-ring resonator can be used in ion chromatography. At a flow rate of 0.5 mL/min, it reaches limits of detection of 485 µM for aspartic acid and 956 µM for lysine.

## 1. Introduction

Split-ring resonators (SRR) are widely used as detectors because they are highly sensitive to changes in permittivity and conductivity of a material placed near the split. In the most basic setup, a split-ring resonator consists of a microstrip line formed to a ring with a split, which is coupled to a second microstrip line. Applications using SRRs are broad including the analysis of organic tissue [1], rotation and distance [2] or detecting cracks in metallic and non-metallic materials [3]. However, the most common use for split-ring resonator-based sensors are biosensors for measuring liquid samples including detecting biological substances such as C-reactive protein (CRP) [4], DNA-hybridization [5], prostate-specific antigen (PSA) [6] and Heparin [7]. An additional application for SRRs is determining the dielectric properties of liquids [8,9,10,11]. Detecting changes in the dielectric properties has specific relevance for chromatographic applications in the liquid phase including high performance liquid chromatography (HLPC), ultra-high pressure liquid chromatography (UHPLC) or ion chromatography (IC) [10,11]. In the design of SRRs, the topologies differ in the structure for coupling of the electromagnetic wave and the form and the size of the ring, which results in different resonance frequencies for the detected medium. Some designs in the literature use an antenna coupling [7,12,13] while others use microstrip lines for coupling [4,8]. The rings are formed either in a circle [7,12,13], quadratic [8,12] or quadratic with cut off edges [4]. Circular split-ring resonators have the advantage that no reflection along the ring occurs. However, the coupling is less with circular SRRs than with quadratic split-rings because the coupling length is significantly smaller. Therefore, the quadratic split-ring with cut-off edges is a good compromise. The split topology significantly influences the sensitivity of the SRR; therefore, an interdigitated structure is often used in the literature as well as in this work [4,14,15,16,17,18]. The frequencies used in the literature are between 100 MHz and 8 GHz [8,12]. Only a small fraction of split-ring resonator based sensors presented in literature comes without any selective coating. Such uncoated split-ring resonators are unselective but highly sensitive detectors for a broad range of compounds and are thus well-suited for application in chromatography [10,11]. In this context, we investigate an uncoated split-ring resonator for the detection of amino acids in liquids. 

Amino acids are small molecules of high biological relevance because they are the building blocks of peptides and proteins. They are needed for the growth and maintenance of cells, part of the cytoskeleton, protein components of enzymes, signaling molecules, and receptors [19] (pp. 1–16). However, essential amino acids cannot be produced by the organism and thus must be supplied through the nutrition [20]. Therefore, the quantitative and qualitative analysis of amino acids in food and dietary supplements is important. As amino acids are required in the growth of cells, amino acids are often included as a component in cell culture media. In terms of production, quality assurance and control of the exact composition of the cell culture media regarding the concentration of the various amino acids is an important application [21,22,23]. Unregulated control of concentration of amino acids in these cell culture media can lead to unstable metabolism and irreproducible results which may have dire consequences in clinical applications [24].

Detecting amino acids in UHPLC-MS (mass spectrometry) grade water following separation with an HPLC or IC with a UV/vis (ultraviolet/visible) detector requires derivatization of the amino acids to make them UV active and detectable [25]. However, derivatization requires additional work and is also unfavorable in cases where the sample is undergoing further analysis or synthesis following chromatographic separation. The objective of this work is to show that a split-ring resonator can detect amino acids without derivatization to avoid additional sample preparation and allow for further analysis and synthesis of the unaltered sample.

## 2. Materials and Methods

**Figure 1:**
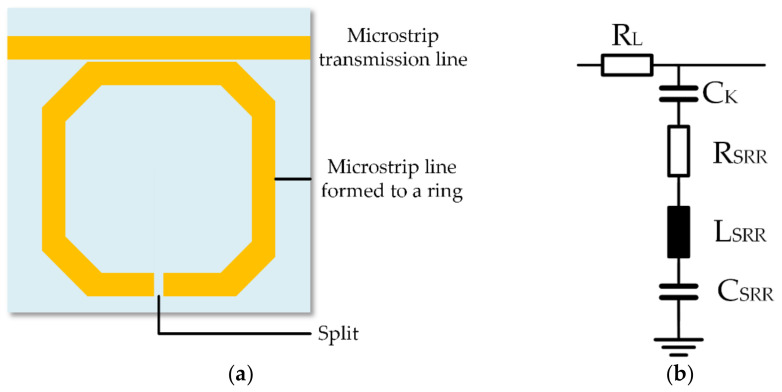
(**a**) Schematic of a split-ring resonator consisting of a printed circuit board with two microstrip lines, one formed to a ring with a split; (**b**) equivalent circuit of the SRR with the ohmic resistance of the transmission line *R_L_*, the coupling capacitance *C_K_*, the ohmic resistance of the ring *R_SRR_*, the inductance of the ring *L_SRR_* and the capacitance of the ring *C_SRR_*.

Split-ring resonators are a type of RLC series resonant circuit built from a microstrip transmission line and a ring structure with a split, shown in Figure 1. At resonance frequency, the wave couples into the ring and forms a standing wave leading a high electric fields inside the split. Therefore, the resonance frequency of split-ring resonators can be calculated with Equation (1) and is accordingly dependent on the inductance *L_SRR_* and the capacitance *C* [8,12]. Considering additional losses, such as ohmic losses, polarization losses at the split or dielectric losses of the printed circuit board, the resonance frequency can deviate from Equation (1). Polarization losses are energy losses caused by polarizing the dielectric. Such polarization losses depend on the excitation frequency and can be represented by an ohmic resistor with frequency dependent value in parallel to the capacitor, but need to be distinguished from ohmic losses caused by a dielectric with a certain conductivity. Therefore, polarization losses damp the resonant circuit as ohmic losses damp the resonant circuit:

(1) $$f_{res}=\frac{1}{2\pi \sqrt{L_{SRR}C}}\;\mathrm{where}\;C=\frac{C_{K}\;\cdot \;C_{SRR}}{C_{K}+C_{SRR}}$$

The SRR is modeled by the equivalent circuit provided in Figure 1b. The transmission line is represented by the ohmic resistance *R_L_*. The capacitive coupling between the two microstrip lines is represented by the coupling capacitance *C_K_*. The microstrip line formed to a ring is an RLC series resonant circuit consisting of the capacitance of the ring *C_SRR_*, the inductance of the ring *L_SRR_* and the resistance of the ring *R_SRR_*. Here, the capacitance *C_SRR_* includes the capacitance of the split and the parasitic capacitance between the ring and the ground plane. The *R_SRR_* includes the ohmic and polarization and dielectric losses, respectively. A detailed description is given by Lippmann et al. [26]. The inductance *L_SRR_* is determined in particular by the shape and size of the ring and can thus be assumed constant in a given design. The capacitance *C* is composed of the capacitance between the two microstrip lines, the capacitance between the microstrip lines and the ground plane, and the capacitance of the split. Only the capacitance of the split is variable by changing the sample at the split. The other capacitances are assumed to be parasitic capacitances *C_p_.* These capacitances are material and frequency dependent. Since the substrate material remains constant and also the used frequencies are constant for each measurement, *C_p_* remains constant. A change of the capacitance *C* affects the resonance frequency. This can be caused by a change of the sample composition at the split and thus the sample’s permittivity, conductivity or polarization losses and is used in many applications for detection of changing sample composition. In addition to the resonance frequency, the Q-factor of the resonant circuit can also change. The Q-factor is also affected by changes in conductivity and polarization losses, but not permittivity. The Q-factor *Q* can be calculated using Equation (2) that shows that it is also influenced by the inductance *L_SRR_* and the capacitance *C*. A high resistance *R_SRR_* caused by low conductivity of the used materials or high dielectric losses of the sample at the split leads to high bandwidth and low Q-factor *Q* [8]:

(2) $$Q=\frac{1}{R_{SRR}}\cdot \sqrt{\frac{L_{SRR}}{C}}$$

Transmission line theory provides another relation between the real part of the permittivity of the sample and the resonance frequency. Assuming that the ring corresponds to a dipole antenna with capacitive prolongation, Equation (3) is obtained by Reinecke et al. including the capacitance [4]:

(3) $$f_{res}=\frac{v}{2l_{0}+\sqrt{4l_{0}^{2}+16l_{0}vZ_{L}\;\cdot \;C}}\;\mathrm{where}\;C=C_{p}+C_{0,p}\;\cdot \;\varepsilon_{r,p}^{\prime }$$

Thus, $f_{res}$ can be calculated from Equation (3) with $l_{0}$ being half of the geometric length of the resonator ring, *v* is the propagation velocity of the wave, *Z_L_* is the impedance of the ring structure that is defined by the geometric parameters especially by the thickness and width of the microstrip line in combination with the ground plane, and *C* is the effective capacitance. The effective capacitance *C* is composed of the parasitic capacitance *C_p_* between the ring and the ground plane and the capacitance of the unloaded (with air) split *C*_0,*p*_ multiplied by the real relative permittivity of the sample $\varepsilon_{r,p}^{\prime }$. A higher permittivity leads to an increase in capacitance and thus to a reduction in the resonance frequency and to an increase in the effective length of the resonator ring [4]. The capacitance *C*_0,*p*_ mainly depends of the distance and the area of the split.

In general, determining a resonance frequency is much easier and more accurate than determining a capacitance. Therefore, we propose a resonant RLC circuit in the form of a simple split-ring resonator with its resonance frequency being an intrinsic value depending on the measured quantity rather than a single capacitor, which however remains the sensitive element in the resonant RLC circuit. The accurate determination of the capacitance of a single capacitor is additionally affected by the measuring device for the determination of the capacitance and the measuring cables required for this method. The effect of external influences, such as mechanical deformation of the cables, has a much more significant impact on the measurement result for determining the capacitance than for determining the resonance frequency with the SRR. In particular, deforming the cables has no effect on the SRR because the components that determine the resonance frequency are mechanically fixed to the PCB.

**Figure 2:**
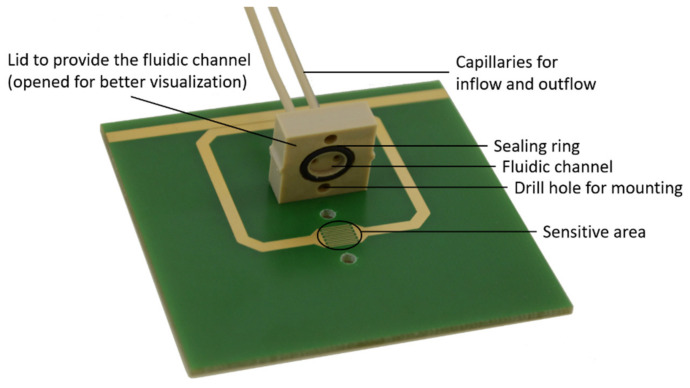
Split-ring resonator consisting of an FR4 printed circuit board with two microstrip lines (the top one for transmitting the wave and the other one is the split-ring micro strip line with the split as interdigital structure as sensitive area) and solder mask with open lid (for better visualization).

**Figure A1:**
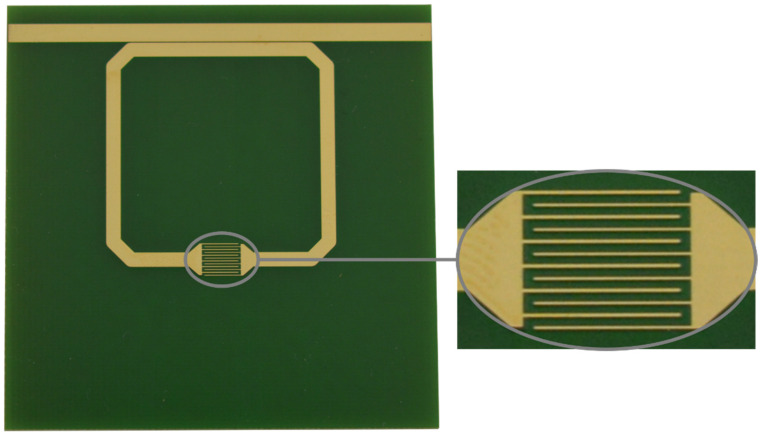
Used SRR with interdigital structure (**left**) with enlarged interdigital structure (**right**) with 6 fingers with a length of 5 mm, 150 µm width and spacing between fingers.

The SRR shown in Figure 2 is used within this work and consists of two microstrip lines on a printed circuit board (PCB) with FR4 as dielectric material and a thickness of 1.4 mm. A detailed explanation of the SRR including simulations and experiments is given in [27]. To prevent liquids from breaking into the dielectric material of the printed circuit board, the surface at the top except the microstrip lines is coated with a solder mask with a thickness of about 25 µm. The first microstrip line is designed for an impedance of 50 Ω adapted to the measurement electronics and is used for coupling the electromagnetic wave in and out of the isolated second microstrip line. The second microstrip line is formed to a quadratic ring with cut off edges with a split. This quadratic topology allows for better coupling to the first microstrip line and less reflection at the edges [4]. The spacing between the two microstrip lines is 100 µm. The spacing between the two microstrip lines is optimized for best coupling between these two for the used frequency range. In addition, 100 µm is the minimum possible spacing between two microstrip lines due to the manufacturing process [28]. The split area is enlarged by using an interdigital structure with 6 fingers with a length of 5 mm, 150 µm width and 150 µm spacing between each other (see Figure A1 in Appendix A). Especially the spacing of the split electrodes and therefore the fingers have a great impact on the penetration depth of the electric field into the sample caused by the coupled electromagnetic wave. A fluidic channel is provided by a PEEK (polyetheretherketone) lid that is screwed onto the PCB. By means of two capillaries, which are connected to the lid, the fluid is fed in and out. On the bottom, the PCB has a ground plane with a void opposite the split capacitor. The microstrip lines and the ground plane are made of 35 µm copper coated with 3 µm to 6 µm nickel and 0.05 µm to 0.1 µm gold on top resulting in a total thickness of 38.05 µm to 41.1 µm.

First measurements of the frequency response are performed with a vector network analyzer (VNWA) Rohde & Schwarz (Munich, Germany) ZNL6. The transmission over the microstrip line (S_21_-parameter) and therefore the attenuation of the SRR is measured over a broad frequency range. The settings of the vector network analyzer are a frequency range of 100 to 400 MHz with a point spacing of 50 kHz. This broad frequency range was carefully chosen to catch all relevant features in the frequency spectrum, in particular, the full resonance peak to determine the exact resonance frequency shift and Q-factor of the SRR even for samples that cause significant damping and resonance frequency shifts. The used measurement frequency range has no influence on the sensitivity of the measurement, since the used VNWA has an equal point spacing also with the broad frequency range. With a bandwidth of 100 Hz and an output power of 0 dBm, a sweep time of 50 s is determined for the set frequency range. Thus, it is only possible to record a spectrum every minute. In addition, the sample at the split must remain constant over the entire sweep time in order to obtain a valid result. Therefore, with this measurement setup, measurements are only possible in static mode. To avoid any fluidic effect during the frequency sweep, we stopped the flow after filling the device with sample liquid. The advantage of this measurement method is that not only the resonance frequency but also the Q-factor of the resonant circuit can be determined from the measurement of the frequency response over the set frequency range. This leads to a measurement sequence in which UHPLC-MS grade water is first injected into the split, followed by the measurement of the frequency response. Afterwards, UHPLC-MS grade water with various concentrations of single amino acids are injected one after the other, and a frequency sweep to measure the frequency response per amino acid and concentration is performed. Finally, the data sets are transferred to the PC and subsequently analyzed with a Matlab^®^ script without any filtering. The raw data are imported into Matlab and the frequency at the minimum of the attenuation is determined as the resonance frequency. Based on the sweep with water, the frequencies are determined for evaluation at a fixed frequency. These are located in the minimum as well as in the turning point of the resonance peak. For all measurements, the values of the attenuation at these frequencies are then determined. The Q-factor is determined from the bandwidth at half attenuation in the resonance peak *b* and the resonance frequency following Equation (4):

(4) $$Q=\frac{f_{res}}{b}$$

**Figure 3:**
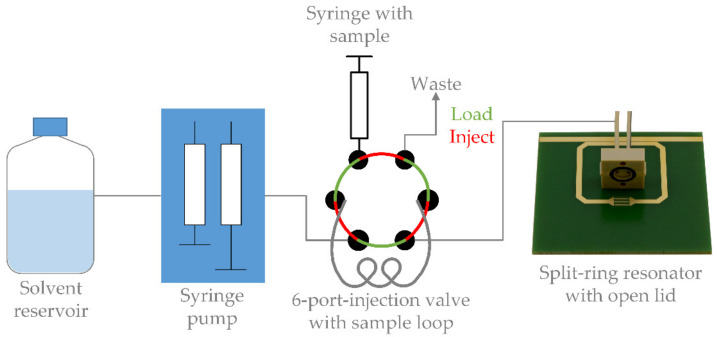
Measurement setup for continuous flow consisting of a solvent reservoir, two syringe pumps to achieve continuous flow, 6-port-valve with sample loop with a volume of 20 µL for sample injection and the split-ring resonator with open lid (for better visualization).

A broadband frequency sweep is time consuming and thus not suitable for recording continuous processes. Therefore, further experiments with a continuous flow rate of 0.5 mL/min are performed with the setup shown in Figure 3, while measuring the amplitude continuously with an envelope detector at a constant measuring frequency. The continuous flow rate is provided by two syringe pumps of the manufacturer Cetoni (Korbussen, Germany) Nemesys S. To the continuous flow rate of 0.5 mL/min UHPLC-MS grade water, 20 µL of a sample (UHPLC-MS grade water with various concentrations of single amino acids) introduced via a sample loop are added using a 6-port valve. For this purpose, the flow is switched, instead of flowing directly from the pump through the valve to the SRR, the flow is re-routed from the valve through the sample loop, through the valve again and finally to the SRR. This ensures that neither the flow nor the pressure is changed or even interrupted. Furthermore, the time and exact amount of sample passing through the SRR are known. With a detector volume of 23 µL, a sample volume of 20 µL and a flow rate of 0.5 mL/min, the sample volume passes through the detector in 2.3 s.

The measurements are performed with an envelope detector that measures the attenuation with a maximum possible sampling rate of up to 5.4 kS/s. In our experiments, a sampling rate of about 1350 Hz including averaging four measuring points is used. The envelope detector has a high frequency stability with a maximum frequency delta of about 840 mHz. The amplitude is also very stable with a maximum delta of about 0.0022 dBm [10]. To determine the optimum measuring frequency with this setup, a broad frequency sweep comparable to that of the vector network analyzer is performed prior to each measurement. The used sweep characteristics are 100 MHz to 350 MHz, 100 kHz point spacing and a power of 5 dBm. All measurements are performed in UHPLC-MS grade water (Sigma product: 1.03728) at pH 7. L-amino acids are used because these are proteinogene and therefore compounds of cells [24]. All chemicals that were used for the experiments were purchased from Sigma-Aldrich, Darmstadt, Germany, with a purity of >98%, L-aspartic acid (Sigma product: A8949), L-glutamine (Sigma product: 49419), L-glutamic acid (Sigma product: G1251), glycine (Sigma product: G7126), L-histidine (Sigma product: H8000), L-isoleucine (Sigma product: I2752), L-lysine (Sigma product: 62840), L-methionine (Sigma product: M9625) and L-threonine (Sigma product, T8625).

## 3. Results

### 3.1. Evaluation Method

**Figure 4:**
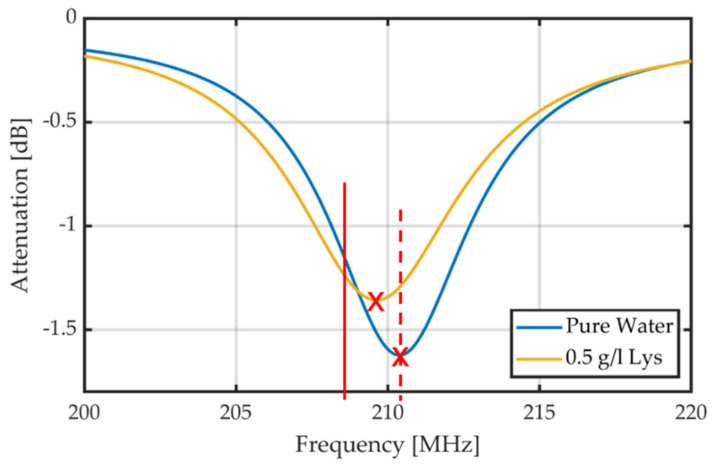
The three different evaluation methods visualized for 0.5 g/L Lysine (Lys) in UHPLC-MS grade water and UHPLC-MS grade water. 1. Tracking the resonance frequency is marked with a red crosses. 2. Measuring the attenuation at the fixed frequency defined by the resonance frequency for UHPLC-MS grade water is marked with a red dashed line. 3. Measuring the attenuation at the fixed frequency defined by the point with the highest slope with UHPLC-MS grade water is marked with a red solid line.

The first three different evaluation methods (resonance frequency vs. attenuation at two different fixed frequencies) are investigated. Therefore, the frequency response is measured over a broad frequency range and the three different evaluation methods, visualized in Figure 4, are compared. The first principle for evaluation uses the resonance frequency as a measure for the concentrations of amino acids. For this purpose, the minimum attenuation is determined from the frequency sweep (marked with red crosses in Figure 4). The other principles for evaluation use the attenuation at a fixed frequency. These two frequencies are determined from the frequency sweep with UHPLC-MS grade water. One frequency is set to the resonance frequency for UHPLC-MS grade water (shown in Figure 4 with a red dashed line). The other frequency is set to the turning point of the resonance peak at lower frequency defined as the frequency with the highest slope in attenuation for UHPLC-MS grade water (marked in Figure 4 with a red solid line). This point is determined as the best frequency when the sample mainly affects the resonance frequency [10].

**Figure 5:**
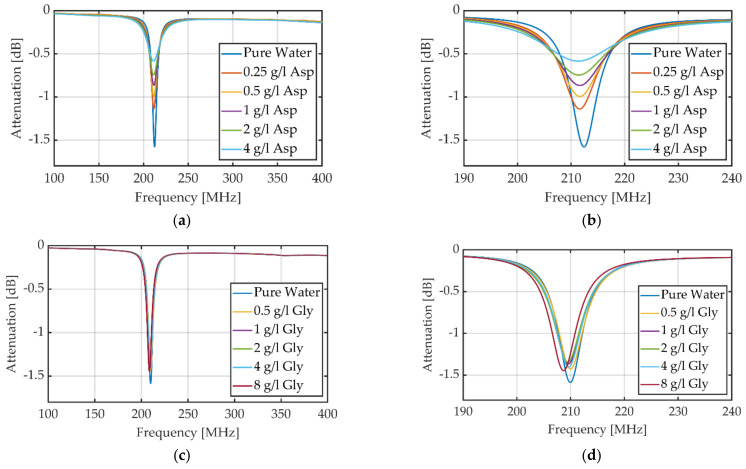

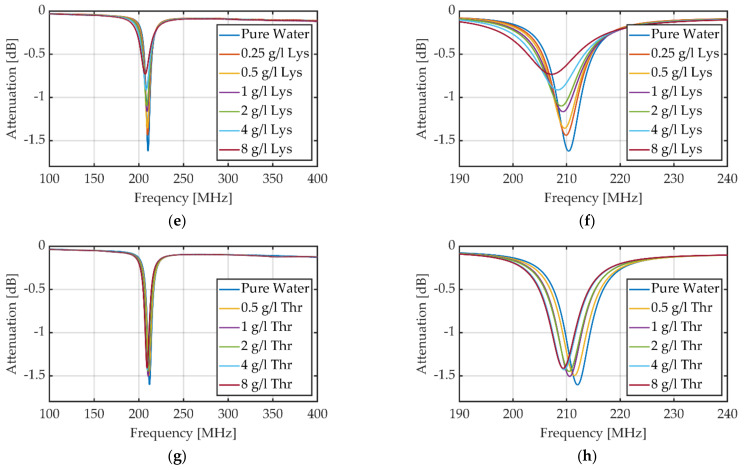
Frequency responses for different concentrations of aspartic acid (Asp) (**a**,**b**), glycine (**c**,**d**) lysine (Lys) (**e**,**f**) and threonine (Thr) (**g**,**h**). The left diagrams show the full frequency spectrums from 100 MHz to 400 MHz for the different concentrations of the four samples. The right diagrams show the same measurements zoomed in to 190 MHz to 240 MHz for better visualization.

If the sample at the split mainly changes the attenuation in case of resonance, the resonance frequency for UHPLC-MS grade water is expected to be the frequency with the highest sensitivity. To determine the sensitivity of each evaluation principle for amino acids, the measurements, shown in Figure 5, performed with the VNWA with four different model amino acids (aspartic acid, glycine, lysine and threonine) are used. These four amino acids have different characteristics. For example, aspartic acid has a negative charged and polar side chain when dissolved in water. Therefore, it increases the electrical conductivity and thus affects the quality factor of the resonant circuit [29]. Lysine has a positive charged and polar side chain when dissolved in water. Therefore, it should also affect the quality factor of the split-ring resonator [30]. In contrast to aspartic acid and lysine, threonine should affect the quality factor less because its side chain is polar but uncharged [19] (pp. 10–14) [30]. Glycine is the simplest and smallest amino acid. It is non-polar and has an aliphatic side chain and therefore should affect the resonance frequency as well as the Q-factor less than the other model amino acids.

The sensitivities shown in Table 1 are used to calculate the limits of detection given in Table 2 under consideration of the noise. The noise for all evaluation methods is determined from 30 measurements with UHPLC-MS grade water. The noise using the tracking resonance frequency method (marked with red crosses in Figure 4) is 66.87 kHz. Using the attenuation at fixed frequency that is the resonance frequency for UHPLC-MS grade water (marked with a red dashed line in Figure 4), the noise is 0.004 dB. With the evaluation method using the attenuation at fixed frequency that is the left turning point of the frequency response for UHPLC-MS grade water (marked with a red solid line in Figure 4), the noise is 0.015 dB. The VNWA was calibrated using the TOSM (through, open, short, match)-method. The Rohde & Schwarz (Munich, Germany) ZNL6 used has a typical noise level of −140 dB normalized for 1 Hz for a bandwidth of 1 kHz and a frequency range from 50 MHz up to 4.5 GHz. The output power has a typical accuracy of 0.5 dB (at a source power of −10 dBm) and a resolution of 0.01 dB. The temperature stability is specified with 0.03 dB/K [31]. The power reference quantity is 0 dBm and therefore 1 mW.

The results, presented in Table 2, show that measuring the attenuation at a fixed frequency that is the resonance frequency for UHPLC-MS grade water at the split leads to the best limits of detection. Both other methods result in an order of magnitude higher limits of detection. Because this method gives the best performance, the method of using the attenuation at fixed frequency that is the resonance frequency for UHPLC-MS grade water is used to evaluate all following measurements.

### 3.2. Measuring Amino Acids

The limits of detection of nine different amino acids are determined using the attenuation method at fixed frequency that is the resonance frequency for UHPLC-MS grade water. First, the full frequency spectra are measured with the amino acids in different concentrations. For aspartic acid, shown in Figure 5a,b, it is obvious that the resonance frequency does not differ significantly, but the attenuation at resonance frequency increases from −1.605 dB to −0.582 dB with increasing concentration. Aspartic acid increases the electrical conductivity and therefore the losses of the split capacitor because of its polar and negative charged side chain and its acidic behavior [24,29]. The evaluation via attenuation at fixed frequency leads to a sensitivity of 97.49 dB/M and considering the noise of 0.004 dB to a limit of detection of 122.50 µM. The noise stays the same for all following measurements with zero flow rate performed with the VNWA. 

With increasing concentration of glycine in UHPLC-MS grade water, shown in Figure 5c,d, the resonance frequency decreases slightly from 210.7 MHz to 208.3 MHz. The attenuation also decreases from −1.546 dB for UHPLC-MS grade water to −0.938 dB for 8 g/L glycine in UHPLC-MS grade water caused by dielectric losses of the split capacitor. The sensitivity for glycine in UHPLC-MS grade water is determined to 12.21 dB/M. Accordingly, the limit of detection is 992.21 µM considering the noise. An increasing concentration of lysine decreases the resonance frequency slightly from 210.4 MHz for UHPLC-MS grade water to 207.2 MHz for 8 g/L lysine in UHPLC-MS grade water; this is shown in Figure 5e,f. The attenuation also decreases significantly from −1.623 dB to −0.559 dB because of the polar and positive charged side chain and the basic behavior of lysine [24]. This leads to a sensitivity of 39.33 dB/M and accordingly to a limit of detection of 308.17 µM. Next, threonine is dissolved in UHPLC-MS grade water and measured. The results are shown in Figure 5g,h. For this amino acid, the resonance frequency decreases slightly with increasing concentration from 212 MHz to 211.3 MHz and attenuation at resonance frequency decrease slightly with increasing concentration from −1.605 dB to −0.884 dB. This can be explained because threonine belongs to the group of amino acids with polar uncharged side chains. The resonance frequency and attenuation of UHPLC-MS grade water differs slightly between the different measurements because of different temperature, bending of the coaxial cables and electromagnetic interference.

**Figure A2:**
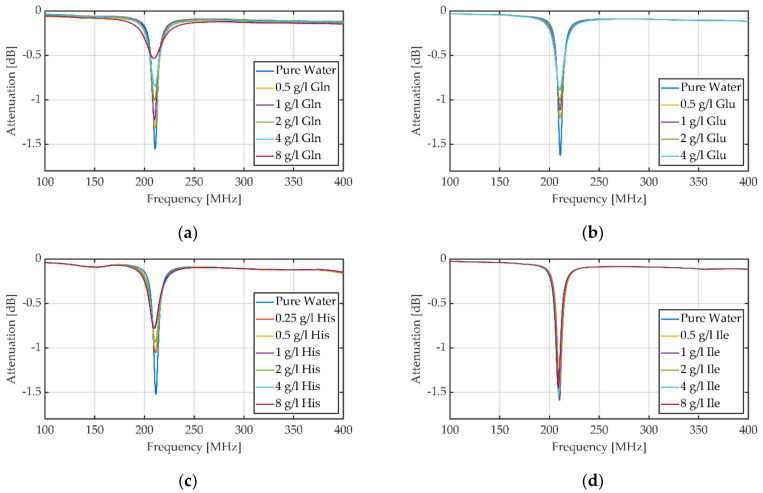

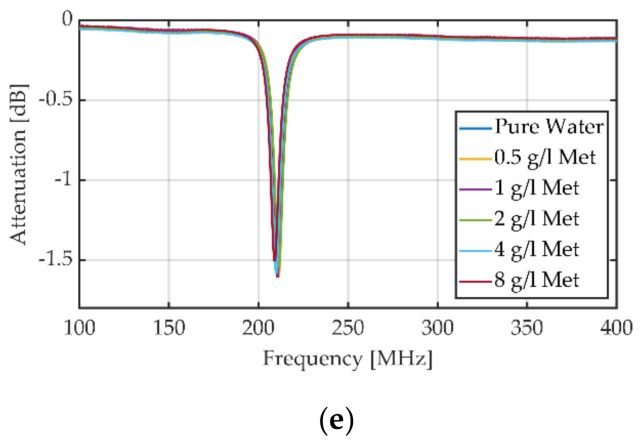
Frequency responses for different concentrations of glutamine (Gln) (**a**), glutamic acid (Glu) (**b**), histidine (His) (**c**), isoleucine (Ile) (**d**), and methionine (Met) (**e**) between 100 MHz and 400 MHz.

The limits of detection of the other amino acids are determined to 229.38 µM (His) (see Figure A2c), 1563.28 µM (Ile) (see Figure A2d), 624.50 µM (Gln) (see Figure A2a), 1504.06 µM (Met) (see Figure A2e) and 105.44 µM (Glu) (see Figure A2b) by measuring the attenuation at the fixed frequency that is the resonance frequency when the split-ring resonator is loaded with UHPLC-MS grade water in static mode. All sensitivities and limits of detection are summarized in Table 3.

How amino acids affect the dielectric properties at the split capacitor strongly depends on their properties, decisive for their detection. An important characteristic for the detection of amino acids is the polarity. Polar amino acids can be detected with better limits of detection than non-polar amino acids because the polar molecules cause higher dielectric losses of the split capacitor. For the polar amino acids (Asp, Glu, Gln, His, Lys, Thr), the limits of detection correlate well will with the isoelectric point. The measurements are performed at pH 7, which means, if the isoelectric point of the amino acid is 7, the positively and negatively charged ions precisely balance with each other. A low isoelectric point means that there are many negative charged ions in the solution, while a high isoelectric point means that there are many positive charged ions. The larger the offset from 7, the better the limits of detection. Only histidine does not fit into this explanation, which can be explained by its aromatic structure and therefore free pairs of electrons that cause higher dielectric losses and higher attenuation at the measuring frequency.

**Figure 6:**
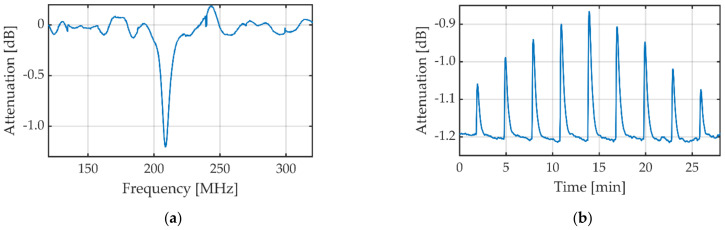

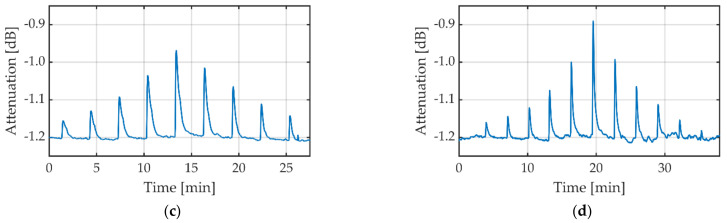
(**a**) Frequency spectrum for UHPLC-MS grade water for determining the resonance frequency and measuring frequency, respectively. Continuous measurements of aspartic acid (**b**), glutamic acid (**c**), and lysine (**d**) at different concentrations in UHPLC-MS grade water. The attenuation for all attenuation plots (**b**–**d**) is median and low-pass filtered.

As already mentioned, chromatographic applications need continuous measurement of the electromagnetic properties of the eluent [10]. Therefore, the limits of detection of three different amino acids are measured with a continuous flow of 0.5 mL/min with the envelope detector described above. With a sample loop of 20 µL, an ideal temporal plug of 2.4 s results, for the real temporal plug, diffusion has to be considered. Following Section 3.1, the measuring frequency is set to the resonance frequency for UHPLC-MS grade water. To determine the measuring frequency, a broad frequency sweep, shown in Figure 6a similar to that with the vector network analyzer, is performed. In this sweep, the minimum is determined and the corresponding frequency of 208.7 MHz is used as measuring frequency for the following measurements. The attenuation is measured continuously at this measuring frequency. First, 20 µL of different concentrations of aspartic acid are injected into the 0.5 mL/min UHPLC-MS grade water flow using the 6-port-valve. The used concentrations are 0.5 g/L, 1 g/L, 2 g/L, 3 g/L and 4 g/L. The concentrations were measured in a systematically increasing and then decreasing manner. The resulting curve in Figure 6b that is median and low pass filtered shows that the height of the peak corresponds well to the concentration of aspartic acid. The determined sensitivity for low concentrations is 235.90 dB/M. Considering the noise of 0.0382 dB with 1 ms of averaging, the limit of detection is 485.35 µM. The same measurement is provided for glutamic acid in UHPLC-MS grade water; see Figure 6c. For glutamic acid, the concentrations are 0.25 g/L, 0.5 g/L, 1 g/L, 2 g/L and 4 g/L (measured up and back down). The sensitivity for low concentrations is 186.00 dB/M, which leads to a limit of detection of 615.59 µM considering the noise. In Figure 6d, the same measurement is shown for lysine in UHPLC-MS grade water. Here, the concentrations are 0.25 g/L, 0.5 g/L, 1 g/L, 2 g/L, 4 g/L and 8 g/L (measured up and back down). The sensitivity for low concentrations is 129.80 dB/M, which leads to a limit of detection of 882.51 µM considering the noise.

With a substrate material with less dielectric losses, the SRR can be further improved. The topology of the SRR can also be optimized to achieve higher quality factors and higher sensitivities by adapting the interdigital structure, the size of the ring and the spacing between the ring and the transmission line. Thus, the limits of detection can be further improved.

## 4. Discussion

The objective of this work is just to present the fundamentals of using a split-ring resonator as a new detector in ion chromatography with amino acids as model substances. Unfortunately, coupling the split-ring resonator to ion chromatography has not been realized yet so that this is not covered in the publication. Therefore, we cannot say much about future applications and have not approached clinical partners yet. The limits of detection of amino acids depend on the properties of the side chains of the amino acids. For example, amino acids with polar and charged side chains (Asp, Glu, His, Lys) have the lowest (best) limits of detection (starting at 105.44 µM for glutamic acid). Especially for amino acids with isoelectric points far from neutral (basic or acidic), the SRR shows high sensitivities and therefore good limits of detection. In contrast, amino acids with nonpolar and aliphatic side chains (Gly, Ile, Met) have the highest limits of detection (up to 1563.28 µM for isoleucine) of the tested amino acids [30]. The reached limits of detection show that the split-ring resonators can be used to detect all tested amino acids in water. With the help of an envelope detector, continuous recording of concentration at a flow rate of 0.5 mL/min is also possible. Here, the limits of detection are slightly higher than in static mode because the used sample volume of 20 µL is smaller than the volume of the sample chamber of 23 µL. The noise can be reduced by increasing the averaging time and thus also the limits of detection. However, the averaging time is limited by the temporal width of the plug eluting from the IC. The advantage of the SRR compared to conventional detectors for the measurement of amino acids such as UV/Vis detectors is that no derivatization is necessary. Therefore, no additional sample preparation is needed and the analytes can be used unaltered for further analysis.

## Figures and Tables

**Table 1:** Sensitivities of aspartic acid, glycine, lysine, and threonine measured with the vector network analyzer (tracking the resonance frequency shift vs. recording the attenuation at two different fixed measuring frequencies, the resonance frequency and the left turning point of the frequency response for UHPLC-MS grade water). * M is defined as mol/L, dB is measured at a power of 0 dBm, and thus the power is referenced to 1 mW.

Amino Acid	Sensitivities of Different Evaluation Methods		
	Tracking Resonance Frequency [MHz/M]	Attenuation at Fixed Frequency [dB/M] *	
		Resonance	Turning Point
Aspartic Acid	45.27	98.93	12.01
Glycine	20.76	12.21	4.04
Lysine	170.17	39.33	7.45
Threonine	34.81	28.91	7.85

**Table 2:** Limits of detection of aspartic acid, glycine, lysine, and threonine measured with the vector network analyzer (tracking the resonance frequency shift vs. recording the attenuation at two different fixed measuring frequencies, the resonance frequency and the left turning point of the frequency response for UHPLC-MS grade water).

Amino Acid	Limit of Detection [µM] of Different Evaluation Methods		
	Tracking Resonance Frequency	Attenuation at Fixed Frequency	
		Resonance	Turning Point
Aspartic Acid	4431.18	122.50	3721.72
Glycine	9660.91	992.21	11,070.18
Lysine	1178.87	308.17	5996.93
Threonine	5763.30	419.18	5692.34

**Table 3:** Isoelectric points, sensitivities and limits of detection for nine amino acids in UHPLC-MS grade water when measuring the attenuation at the resonance frequency for UHPLC-MS grade water as a measure for amino acid concentration.

Amino Acid	Short	Isoelectric Point [32]	Sensitivity	Limit of Detection
Aspartic Acid	Asp	2.85	97.49 dB/M	122.50 µM
Glutamic Acid	Glu	3.22	115.94 dB/M	105.44 µM
Glutamine	Gln	5.65	47.57 dB/M	624.50 µM
Glycine	Gly	5.97	12.21 dB/M	992.21 µM
Histidine	His	7.47	52.83 dB/M	229.38 µM
Isoleucine	Ile	5.94	7.75 dB/M	1563.28 µM
Lysine	Lys	9.59	39.33 dB/M	308.17 µM
Methionine	Met	5.74	8.06 dB/M	1504.06 µM
Threonine	Thr	5.60	28.91 dB/M	419.18 µM

## Data Availability

The data are available upon request.

## References

[1] Puentes M., Weiß C., Schüßler M., Jakoby R. Sensor Array Based on Split Ring Resonators for Analysis of Organic Tissues. Proceedings of the 2011 IEEE MTT-S International Microwave Symposium.

[2] Naqui J., Durán-Sindreu M., Martín F. (2012). Alignment and Position Sensors Based on Split Ring Resonators. Sensors.

[3] Albishi A., Ramahi O.M. (2014). Detection of surface and subsurface cracks in metallic and non-metallic materials using a complementary split-ring resonator. Sensors.

[4] Reinecke T., Walter J.-G., Kobelt T., Ahrens A., Scheper T., Zimmermann S. (2018). Design and evaluation of split-ring resonators for aptamer-based biosensors. J. Sens. Sens. Syst..

[5] Lee H.-J., Lee H.-S., Yoo K.-H., Yook J.-G. (2010). DNA sensing using split-ring resonator alone at microwave regime. J. Appl. Phys..

[6] Lee H.-J., Lee J.-H., Moon H.-S., Jang I.-S., Choi J.-S., Yook J.-G., Jung H.-I. (2012). A planar split-ring resonator-based microwave biosensor for label-free detection of biomolecules. Sens. Actuators B Chem..

[7] Torun H., Cagri Top F., Dundar G., Yalcinkaya A.D. (2014). An antenna-coupled split-ring resonator for biosensing. J. Appl. Phys..

[8] Withayachumnankul W., Jaruwongrungsee K., Tuantranont A., Fumeaux C., Abbott D. (2013). Metamaterial-based microfluidic sensor for dielectric characterization. Sens. Actuators A Phys..

[9] Ebrahimi A., Withayachumnankul W., Al-Sarawi S.F., Abbott D. (2014). High-Sensitivity Metamaterial-Inspired Sensor for Microfluidic Dielectric Characterization. IEEE Sens. J..

[10] Hitzemann M., Dehning K.J., Gehl A.V., Sterr E.-F., Zimmermann S. (2022). Fast Readout of Split-Ring Resonators Made Simple and Low-Cost for Application in HPLC. Electronics.

[11] Dehning K.J., Hitzemann M., Sterr E.-F., Zimmermann S. P8.9-Split-Ring Resonator as Detector for Liquid Chromatography. Proceedings of the 15. Dresdner Sensor-Symposium 2021.

[12] Verma A., Bhushan S., Tripathi P.N., Goswami M., Singh B.R. (2017). A defected ground split ring resonator for an ultra-fast, selective sensing of glucose content in blood plasma. J. Electromagn. Waves Appl..

[13] Camli B., Kusakci E., Lafci B., Salman S., Torun H., Yalcinkaya A. (2016). A Microwave Ring Resonator Based Glucose Sensor. Procedia Eng..

[14] Ye W., Zhao W.-S., Wang J., Wang D.-W., Wang G. (2022). A Split-Ring Resonator-Based Planar Microwave Sensor for Microfluidic Applications. Proceedings of the 2022 IEEE MTT-S International Microwave Biomedical Conference (IMBioC).

[15] Wu W.-J., Zhao W.-S. (2022). A Quality Factor Enhanced Microwave Sensor Based on Modified Split-Ring Resonator for Microfluidic Applications. IEEE Sens. J..

[16] Yeo J., Lee J.-I. (2019). High-Sensitivity Microwave Sensor Based on an Interdigital-Capacitor-Shaped Defected Ground Structure for Permittivity Characterization. Sensors.

[17] Ye W., Wang D.-W., Wang J., Wang G., Zhao W.-S. (2022). An Improved Split-Ring Resonator-Based Sensor for Microfluidic Applications. Sensors.

[18] Bao X., Zhang M., Ocket I., Bao J., Kil D., Liu Z., Puers R., Schreurs D., Nauwelaers B. (2020). Integration of Interdigitated Electrodes in Split-Ring Resonator for Detecting Liquid Mixtures. IEEE Trans. Microw. Theory Techn..

[19] Schulz G.E., Schirmer R.H. (1979). Principle of Protein Structure.

[20] Fürst P., Stehle P. (2004). What Are the Essential Elements Needed for the Determination of Amino Acid Requirements in Humans?. J. Nutr..

[21] Larson T.M., Gawlitzek M., Evans H., Albers U., Cacia J. (2002). Chemometric evaluation of on-line high-pressure liquid chromatography in mammalian cell cultures: Analysis of amino acids and glucose. Biotechnol. Bioeng..

[22] Kabus P., Koch G. (1982). Quantitative Determination of Amino Acids in Tissue Culture Cells by High Performance Liquid Chromatography. Biochem. Biophys. Res. Commun..

[23] Askretkov A.D., Klishin A.A., Zybin D.I., Orlova N.V., Kholodova A.V., Lobanova N.V., Seregin Y.A. (2020). Determination of Twenty Proteinogenic Amino Acids and Additives in Cultural Liquid by High-Performance Liquid Chromatography. J. Anal. Chem..

[24] Salazar A., Keusgen M., von Hagen J. (2016). Amino acids in the cultivation of mammalian cells. Amino Acids.

[25] Kaspar H., Dettmer K., Gronwald W., Oefner P.J. (2009). Advances in amino acid analysis. Anal. Bioanal. Chem..

[26] Lippmann M., Hermeling L., Hitzemann M., Dehning K.J., Zimmermann S. Electronics for Continuously Measuring the Resonance Frequency and Attenuation of a Split-Ring Resonator. Proceedings of the 16. Dresdner Sensor-Symposium 2022.

[27] Dehning K.J., Hitzemann M., Gossmann A., Zimmermann S. Split-Ring Resonator for measuring low amounts of glutamic acid in pure water. Proceedings of the 16. Dresdner Sensor-Symposium 2022.

[28] Multi-CB Basic Design Rules für Leiterplatten v2.4—05.2021. https://www.multi-circuit-boards.eu/fileadmin/user_upload/downloads/leiterplatten_design-hilfe/Miltu-CB-Leiterplatten_Basic-Design-Rules-pdf.

[29] Apelblat A., Manzurola E., Orekhova Z. (2008). Electrical Conductance Studies in Aqueous Solutions with Aspartic Ions. J. Solut. Chem..

[30] Pethig R. (1984). Dielectric Properties of Biological Materials: Biophysical and Medical Applications. IEEE Trans. Electr. Insul..

[31] Rohde & Schwarz R&S®ZNL Vector Network Analyzer Specifications. https://scdn.rohde-schwarz.com/ur/pws/dl_downloads/dl_common_library/dl_brochures_and_datasheets/pdf_1/ZNL_dat-sw_en_3607-1071-22_v0700.pdf.

[32] Taylor W.R. (1986). The Classification of Amino Acid Conservation. J. Theor. Biol..

